# Recovery of Spent Sulphuric Acid by Diffusion Dialysis Using a Spiral Wound Module

**DOI:** 10.3390/ijms222111819

**Published:** 2021-10-30

**Authors:** Arthur Merkel, Ladislav Čopák, Lukáš Dvořák, Daniil Golubenko, Libor Šeda

**Affiliations:** 1 MemBrain s. r. o. (Membrane Innovation Centre), Pod Vinicí 87, 471 27 Stráž pod Ralskem, Czech Republic; libor.seda@membrain.cz; 2 Institute for Nanomaterials, Advanced Technologies and Innovation, Technical University of Liberec, Studentská 2, 461 17 Liberec, Czech Republic; lukas.dvorak@tul.cz; 3 Kurnakov Institute of General and Inorganic Chemistry of the Russian Academy of Sciences, 31 Leninsky Avenue, 119991 Moscow, Russia; golubenkodaniel@yandex.ru

**Keywords:** anion-exchange homogeneous membrane, membrane degradation, mass balance, case study, payback time

## Abstract

In this study, we assess the effects of volumetric flow and feed temperature on the performance of a spiral-wound module for the recovery of free acid using diffusion dialysis. Performance was evaluated using a set of equations based on mass balance under steady-state conditions that describe the free acid yield, rejection factors of metal ions and stream purity, along with chemical analysis of the outlet streams. The results indicated that an increase in the volumetric flow rate of water increased free acid yield from 88% to 93%, but decreased Cu^2+^ and Fe^2+^ ion rejection from 95% to 90% and 91% to 86%, respectively. Increasing feed temperature up to 40 °C resulted in an increase in acid flux of 9%, and a reduction in Cu^2+^ and Fe^2+^ ion rejection by 2–3%. Following diffusion dialysis, the only evidence of membrane degradation was a slight drop in permselectivity and an increase in diffusion acid and salt permeability. Results obtained from the laboratory tests used in a basic economic study showed that the payback time of the membrane-based regeneration unit is approximately one year.

## 1. Introduction

Industrial processes often generate wastewaters characterized by high acidity or high alkalinity with pH lower than 1 and above 14 respectively in some cases and/or a high metal content [1,2]. Membrane technologies facilitate the recovery of valuable dissolved components such as metals and allow the reuse of acids or alkalis. Among the various membrane technologies presently available, diffusion dialysis (DD), nanofiltration (NF), electrodialysis (ED), membrane distillation (MD) and forward osmosis (FO) are the most promising as regards the circular economy paradigm [3,4]. It is widely believed that DD represents the optimal process for recovery of acids at high concentrations (i.e., >0.5 M) [3]. Compared to other technologies, DD has a number of advantages [5,6], including low energy consumption (due to spontaneous processes driven by activity gradients), low installation costs, simple operation and maintenance, and high product quality (due to the high selectivity of anion-exchange membranes (AEMs) for acids). In addition, the process is considered environmentally friendly due to the lack of post-processing and chemical agents used [3,4,7].

DD is a membrane separation process driven by a concentration gradient and, in environmental engineering, it is mainly used for the recovery of spent acids or alkalis [5,8]. The basic principle of separation utilises the high membrane permeability of acids and alkalis relative to metal salts, in association with proton-transport via the hydrogen bond network of water molecules, a process known as the ‘Grotthuss mechanism’ [9,10,11]. This latter process explains why DD is most often used specifically to recover acids [3]. AEMs are the most frequently used materials for processing of acids via DD. Owing to the positively charged polymer matrix surrounding the pores, diffusion of cations, such as Fe^2+^, Fe^3+^, Cu^2+^ or Pb^2+^ slows down in the AEM-matrix, making it possible to achieve higher selectivity coefficients [6]. In recent years, development of DD has focused on the synthesis of new membranes and modifications to known materials [12,13,14,15], optimization of module structure [16,17], expanding known applications and researching potential new applications [17,18,19,20,21,22].

While laboratory-scale acid recovery experiments using DD are mainly conducted in batch configurations, plant-scale operations benefit from the use of a continuous dialyser, which provides a number of advantages, including higher productivity, lower costs, convenient assemblage and transportation and a continuous process more appropriate for practical production-level use [6,18]. As such, the next step towards advanced DD is usually seen as optimization of the module configuration. At present, ‘plate-and-frame’ is the most commonly used membrane module type; however, the space-saving characteristics and modular nature of spiral-wound DD membrane modules has recently attracted much attention [6,17,23]. Zhang et al. [23], for example, while studying the process of hydrochloric acid separation from aluminium salts using a DD spiral-wound membrane module, showed that acid yield, recovered acid concentration and metal salts rejection were all affected by diffusate and dialysate flow rates. According to these authors, the optimized ratio of feed over water flow rate is around 1. A relative increase in the feed (dialysate) flow led to a decrease in acid yield due to the reduction in solution contact time in the module, while the concentration of recovered acid and leaked aluminium salts increased.

Membrane stability is a key factor for their use in DD, which characterizes the steadiness of the membrane’s physicochemical properties such as permeability and separation factor under operation conditions [6]. In characterizing such membranes, researchers tend to concentrate on the alkaline-stability of AEMs [24], primarily due to their frequent use in alkaline fuel cells. In comparison, there is relatively little data available on the acid-stability of AEMs. Commercial AEMs for DD tend to be more stable than nanofiltration membranes in acid solutions when used for acid recovery based on polyamides [3]. Indeed, it has been reported that commercial and tailor-made AEMs for DD based on bromomethylated poly(phenylene oxide) (BPPO) degrade noticeably in acid solutions within 72 h [25,26], with both loss of mass and reduced ion-exchange capacity reported. Other studies, however, have reported high stability of BPPO-based membranes in acid solutions [27].

In the present study, we assess the capacity of a spiral-wound module designed for continuous recovery of free acid using DD to separate sulphuric acid and Cu^2+^ and Fe^2+^ salts. During this experimental campaign, we also investigate impact of volumetric flow and feed temperature on module performance and assess membrane degradation through characterization of ion-exchange capacity and mechanical and transport properties before and after DD operation.

## 2. Results

### 2.1. Effect of Volumetric-Flow Ratio

**Figure 1:**
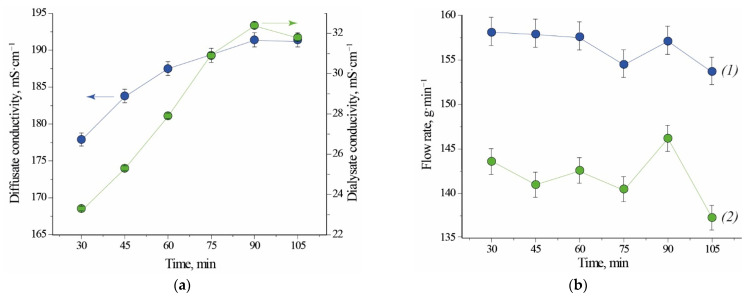
Conductivity (**a**) and flow rate (**b**) as a function of time for Test 1.1. (1) diffusate; (2) dialysate. Initial fill: water. Note that the lines connecting experimental points are not true trendlines.

**Figure 2:**
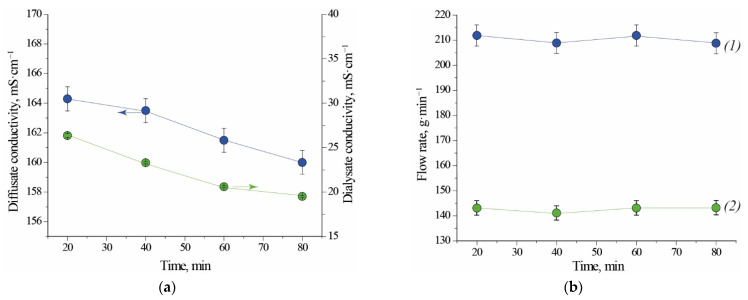
Conductivity (**a**) and flow rate (**b**) as a function of time for Test 2.1. (1) diffusate; (2) dialysate. Initial fill: DD profile from the previous testing. Note that the lines connecting experimental points are not true trendlines.

Electrical conductivity of the outlet streams increases over time for Test 1.1. This behaviour is caused by the fact that diluted acidic solutions have higher conductivity than demineralized water which was present in the module before its run-up. Steady state of the system is visible from the 90th minute of the process on (Figure 1a). In case of Test 2.1, when module was filled with liquid from the previous test, electrical conductivity of both outlet streams decreases over time. From this observation results that acid is recovered with higher efficiency. The electrical conductivity of the regenerated acid is on average 6 to 8 times higher than the electrical conductivity of the processed salty acid stream leaving the module. The stationary electrical conductivity of the diffusate is higher in the case of Test 1.1 (Figure 1a, 191 mS⋅cm^−1^) than in the case of Test 2.1 (Figure 2a, 160 mS⋅cm^−1^). This comparison implies that a volumetric-flow ratio has an impact on the concentration of free acid in the diffusate. A higher flux of acid in the case of Test 2.1 is caused by a higher logarithmic mean of concentration difference (a driving force for mass transfer). For this reason, the mass flow of free acid per exchange-area unit is higher and results in a bigger yield (Table 1). Last two points of dialysate flow rate for Test 1.1 exhibit a relatively big deviation from previously measured values (Figure 1b). Small perturbation could be caused by inaccuracy of the flow valves used for regulation of the inlet feed flowrate. No difficulties were observed with maintaining the set value of flowrate of the inlet streams for Test 2.1 (Figure 2b).

Evidence that the mass transfer occurs in the dialyser can be concluded from the change in the measured flow rates of the inlet stripping medium and the diffusate (Table 2). Preferential transfer of acid is visible from the composition of the streams. In diffusate is present mainly sulphuric acid beside solvent and small amount of iron and copper sulphates. On the contrary, most salts are in dialysate, whereas sulphuric acid is almost not present. Change in composition is also visible in densities of the solutions. Dialysate leaving the module is less dense than feed because of the missing acid (Table 3).

### 2.2. Effect of Feed Temperature

The temperature of the feed for Test 1.2 under laboratory conditions gradually increased (from 22.1 °C at the beginning to 24.4 °C at the end of the test lasting 135 min). This is caused by the dissipation of mechanical energy delivered by the pump. Since part of the pumped feed solution is warmer and recycled back to the feed vessel, it mixes with colder liquid which increases the feed temperature. In the remaining tests (Test 2.2 and 3.2), the set value of temperature was maintained without any issues.

Sulphuric-acid yield does not change much and remains constant over studied temperature range, 76%. The value is low, which is caused by the low flow ratio of input streams (Table 4). Furthermore, this is proof that different volumetric flows of input streams can significantly influence system performance. The measured flow rates again prove that mass transfer occurs in the dialyser and is visible in the increase of the mass flow rate between the inlet RO permeate and the diffusate (Table 5).

If the *R_V_* parameter is higher than 1, then acid yield decreases. On the other hand, the weight fraction of acid in the diffusate increases and approaches the concentration of acid in the feed stream (Table 6). With a higher feed flow rate, the residence time of the dialysate in the module is shorter for acid transfer, so a smaller portion of the fed acid will be recovered. Since stripping medium flows at lower velocity, it will leave the module enriched with an acid (and metal ions as well) with a higher components’ concentration.

### 2.3. Membrane Properties

**Figure 3:**
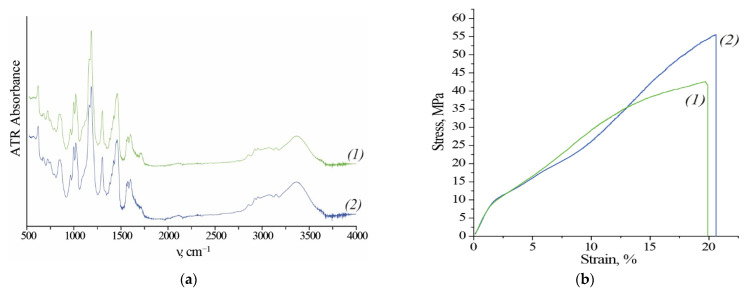
(**a**) Fourier transform infrared spectroscopy (FT-IR) spectra and (**b**) stress-strain curves for membranes (1) before use (initial state) and (2) after use (in a dry state, Cl^−^ form).

According to the manufacturer, the module includes a commercial anion exchange membrane Fumasep^®^ FAD-PET-75 (Fumatech BWT GmbH, Bietigheim-Bissingen, Germany). Fumasep^®^ FAD-PET-75 is a PET-reinforced anion exchange membrane with low resistance, high acid transfer rate, high mechanical stability, and high stability in an acidic environment. After exposition of the membrane to the acidic environment no significant differences in the IR spectra are observed (Figure 3b). Figure 3b shows typical stress-strain curves of membranes before and after use. The curves show the elastic region, yield and strain hardening.

**Figure 4:**
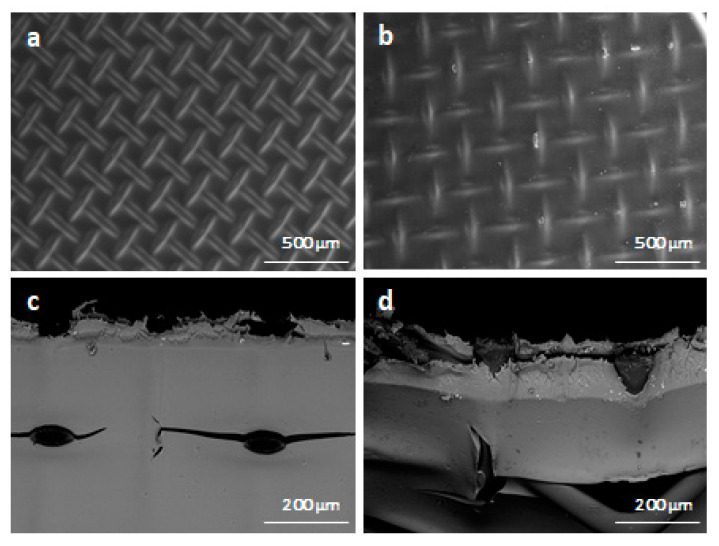
Scanning electron microscopy images detailing membrane state before use ((**a**) surface, (**c**) cross-section)) and after use ((**b**) surface, (**d**) cross-section).

After the operation Young’s modulus, Yield strength and Elongation at break do not change, and the Tensile strength even increases (Table 7). Visually, there are no cracks or traces of degradation of the polyethylene terephthalate (PET) mesh on both SEM images (Figure 4). Most of the physicochemical properties of membranes do not change significantly, but diffusion permeability increases by 22% and permselectivity decrease by 1.4% (Table 7).

### 2.4. Economic Study

**Figure 5:**
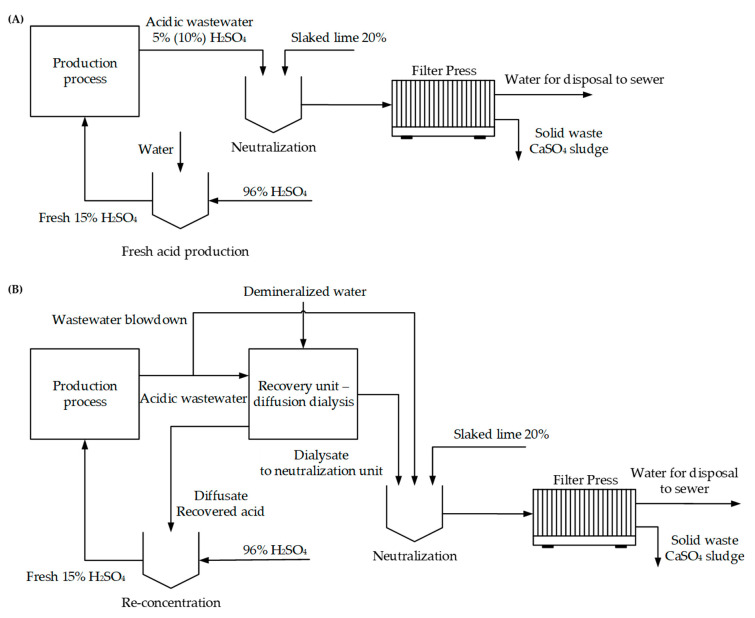
Process flow diagram for the proposed treatment technology. (**A**) treatment of acidic wastewater without diffusion dialysis; (**B**) implementation of dialysis into production-level wastewater management.

The following study deals with expenses and savings before and after implementation of the spiral-wound diffusion dialyzers into a generic plant consuming 15% H_2_SO_4_ and generating acidic waste. The study considers two cases of processing 90 kg·h^−1^ acidic wastewater at various degrees of dilution (5% and 10%) by dialysis. Re-concentration to 15% H_2_SO_4_ is also considered. The treatment section consists of neutralization by 20% suspension of slaked lime (technical-grade purity, 90%) according to a scheme shown in Figure 5A,B.

Solid-liquid suspension after neutralization is then processed in a filter press. The moisture of the solid phase was considered 65%. Economic rentability is based on the usage of 10 modules processing 90 kg/h of acidic feed and on the consumption of 90 kg/h of demineralized water. The yield of acid was considered 88% in the whole range of studied concentrations of H_2_SO_4_ in waste from 5% to 10%. No other salts than CaSO_4_ were considered (treated wastewater did not contain any dissolved salt for the sake of simplicity). In real applications, the metal ions (Fe^2+^, Zn^2+^, Ni^2+^, Cu^2+^, etc.) are precipitated in the hydroxide form out of the solution together with gypsum. Therefore, the mass of solid waste increases proportionally to the concentration of metal ions. To evaluate operating expenses, consumption of electricity of the pumps for the dialyzers, demineralized water for sulphuric acid stripping, 96% sulphuric acid for make-up, slaked lime and disposal of solid waste were taken into consideration. Unit prices of the abovementioned inputs can be found in Table 8. The values can vary from case to case and are only rough numbers. The total power consumption of the pumps of the unit is considered 1 kW. The capital cost of the unit containing 10 modules is around 52,400 USD. In practical applications pre-filtration unit to prevent insoluble particles from penetration into modules is required and its costs need to be considered. The time allowance for the calculation is 330 working days per year and 24 h per day.

Regardless of the studied case, implementation of the recovery unit will lead to significant savings of neutralization agent, concentrated sulphuric acid and money for sludge disposal. Savings of sources, both material (sulphuric acid) and financial, are visible in a summary of operational expenditures (Table 9). Despite 88% of acid recovery, saving of neutralization agent needed to treat not only dialysate but also blowdown stream is 74%.

**Figure 6:**
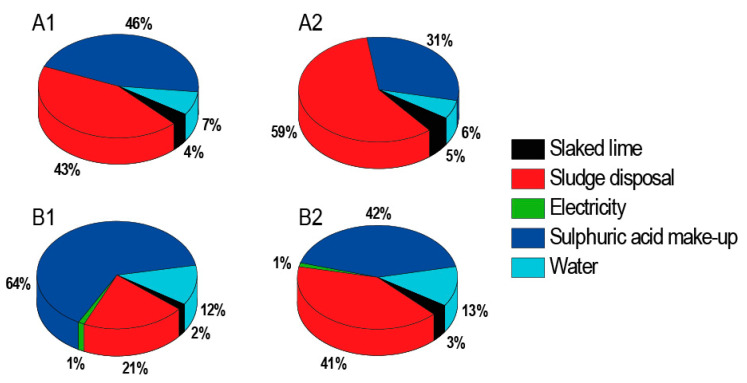
Percentage distribution of operating expenses: (**A1**) processing 5% H_2_SO_4_ wastewater without DD; (**B1**) processing 5% H_2_SO_4_ wastewater with DD; (**A2**) processing 10% H_2_SO_4_ wastewater without DD; (**B2**) processing 10% H_2_SO_4_ wastewater with DD.

From the graphs, it is evident that a significant part of costs is represented by sludge disposal (Figure 6). It is therefore important to pay attention to the maximal possible removal of moisture. Economic calculations have shown that simple payback time can be less than one year and is a strong function of the acid concentration in the treated feed entering the recovery unit. The more dilute acid is processed, the longer is the payback period (Table 10).

## 3. Discussion

The higher the flow rate of the stripping medium, the higher acid yield will be achieved, and the more diluted diffusate will be obtained (cf. Table 1). This inherently leads to a higher consumption of deionized water. Electrical conductivity is a function of concentration and composition. In the case of pure, diluted sulphuric acid solution, electrical conductivity is linearly dependent on an acid concentration. Moreover, protons exhibit high mobility, therefore, acidic solutions have high electrical conductivity. Based on this physicochemical knowledge we can assume how much is the diffusate rich in acid.

The yield of sulphuric acid can be approximately 90%, which also means saving roughly 90% of sources in the neutralization step. Also, by implementing DD the amount of neutralized salt will be decreased by ~90%. The advantage of employing a counter-current configuration of stream flows can be deduced from the obtained results. The concentration of the recovered acid is close to the initial feed value (cf. Table 1). Therefore, an extensive reconcentration step after DD will not be necessary for many applications. Metal cations are retained mainly in the dialysate due to repulsion of cationic species by AEMs with a small concentration of free acid, less than 1 wt. %.

After evaluation of data obtained from analytical analyses, it was found out that the temperature of the feed has an effect on the module performance (cf. Table 4) up to 30 °C. However, this effect is not as significant as the effect of a volumetric-flow ratio. The yield of the acid increased, which is following increased sulphuric acid mass flux through the membranes. Although, at the expense of lower rejection factors of metal ions. This observation can be explained by the relaxation of the membranes. Higher temperatures could modify the internal structure [28,29] of the AEM in a way that the membranes became more permeable for each solute present in the solution. Also, increasing flows can be attributed to the fact that diffusion is the activation process that is accelerated with increasing temperature. Moreover, at temperatures higher than 30 °C, temperature plays a minor role in a mass transfer.

Concentrations of free acid and salts in the wastewater can vary from case to case. In this experimental study, highly acidic conditions (acidity ~1 M) and somewhat moderate conditions in terms of metal ion concentration (<1 g·L^−1^) were considered. Obtained results showed that the yield of sulphuric acid was in the case of unit flowrate ratio 88%. However, it was reported [18] that salts can enhance a mass transfer of a free acid what consequently results in its higher yield.

The physicochemical properties change indicates the high chemical and mechanical stability of membranes. The only evidence of degradation is a slight drop in membrane selectivity and an increase in diffusion permeability. This is a good result relative to other types of membranes, for membranes based on bromomethylated poly (phenylene oxide) (BPPO) degradation manifested in a drop of ion-exchange capacity [25,26].

The payback period can be influenced by several phenomena. Expenses of the cleaning-in-place procedure were excluded from the study since modules unlike RO modules and at good feed quality (no oil, suspended solids, surfactants, or detergents), do not require cleaning. Costs for replacements of the safety AC filters were also excluded from the calculations because pre-filtering will be dependent on the individual cases. Another parameter that is normally measured before release into surface water is dissolved solids. The limit value varies with location and is not the same for each company. In the case of neutralization of wastewater containing sulphuric acid, low soluble calcium sulphate is generated. Its solubility at 25 °C is 2.6 g·L^−1^. From this point of view, emission of such water usually should not lead to fees due to high total dissolved solids. However, the situation is more complicated with wastewater containing HCl, since neutralization of this acid generates very soluble CaCl_2_. For this reason, recovery of HCl should have its place in wastewater management instead of simple neutralization. However, the rejection factor for some metal ions like Zn^2+^ in dialysis of HCl wastewater is lower due to the formation of negatively charged chloro complexes [18], which are not repulsed by the fixed groups and therefore diffuse through the AEM easily. Nevertheless, each case should be assessed separately with a thorough economical study. Actual unit costs should be used to clarify outcomes and benefits after the DD implementation.

## 4. Material and Methods

### 4.1. Feed Solution

A 25 L model solution consisting of sulphuric acid with impurities of iron and copper sulphate was used for each experiment, with a fresh model solution being prepared for each continuous test. The model solution comprised 0.732 L of 96% sulphuric acid (Penta s.r.o., p.a.), 37.3 g of iron(II) sulphate heptahydrate (Penta s.r.o., pure), 4.9 g of copper (II) sulphate pentahydrate (Penta s.r.o., p.a.) and reverse osmosis (RO) permeate (қ < 10 µS·cm^−1^). When made up to 25 L with demineralized water, the resulting solution comprised ca. 5 wt. % of sulphuric acid, 300 ppm of Fe^2+^ and 50 ppm of Cu^2+^ (Table 11). The electrical conductivity of the feed solution ranged from 215 to 231 mS·cm^−1^ at 25 °C.

### 4.2. Analytical Methods

Determination of composition was undertaken via potentiometric alkalimetry for measuring free acid content and inductively coupled plasma (ICP-OES) coupled with optical emission spectroscopy (both Thermo Fischer Scientific GmbH, Bremen, Germany) for measuring metallic ion concentration by a method of a calibration curve. Standard samples had concentrations of 0.1; 0.5; 1; 5; 10 and 100 ppm.

The main parameters which were measured during acid-recovery experiments were electrical conductivity, temperature, density, volumetric flow, and mass flow. Electrical conductivity and temperature were determined using a TetraCon 925/LV-P probe (Xylem Analytics WTW, Germany) connected to a WTW 3430 Multimeter (Xylem Analytics WTW, Germany). Density was measured with a portable hand-held Densito 30PX density meter (Mettler Toledo, Japan). A KERN 572 balance (KERN & SOHN GmbH, Balingen), cylinder and a timer were used for determination of mass flow.

### 4.3. Equipment

A WD-AR10-2001 spiral-wound module (Spiraltec GmbH, Germany) with an effective anion exchange area of ca. 5 m^2^ was used to test the effects of flow rate ratio and temperature on recovery of spent sulphuric acid by DD (Table 12).

### 4.4. Membrane Characterization

CuSO_4_ diffusion permeability was determined from the CuSO_4_ flow with the membrane placed between the 0.1 and 0.001 M CuSO_4_ solutions. Measurements were conducted at room temperature (24 °C) in a two-section plexiglass cell with a 5 cm^2^ active membrane area, the solutions being constantly stirred at ca. 400 rpm by a tailor-made two-position magnetic stirrer. CuSO_4_ flow was determined using an Expert-002 conductometer (Econix-Expert, Russia). Using a similar method, sulphuric acid diffusion permeability was determined from the H_2_SO_4_ flow with the membrane placed between the 0.1 M Na_2_SO_4_ and 0.1 M H_2_SO_4_ solutions. H_2_SO_4_ flow was calculated from pH change in salt solution determined using an Expert-pH pH-meter (Econix-Expert, Russia).

AC-membrane ionic resistance was determined via the four-electrode method, using a thermostatic 25 °C cell filled with a 0.5 M NaCl solution and a P-40X potentiostat-galvanostat with an FRA-24M impedance measurement module (Elins, Russia). A detailed description of the experiment can be found elsewhere [31].

Permselectivity was characterized by the potentiostat technique, the membrane being placed between 0.1 M and 0.5 M NaCl solutions in a two-compartment cell. A detailed description of the experiment and calculations can be found elsewhere [32].

Stress-strain experiments were performed using a Tinius Olsen H5KT universal testing machine and a force sensor set at 100 N under ambient conditions (24 °C/25% relative humidity). The gauge length of the samples was adjusted to 50 mm for the machine and the strain rate was set at 5 mm/min for all tests. A more detailed description of the experiment can be found elsewhere [33].

Water uptake was determined from weight loss after drying the film at 80 °C for several hours. Dimensional swelling was determined by the change in geometric dimensions of the membrane sample before and after dehydration.

Scanning electron microscope (SEM) images were obtained using a Quanta FEG 450 SEM (FEI, Hillsboro, OR, USA) at 10kV accelerating voltage and 80 Pa residual pressure. A detailed description of ion exchange capacity measurement and calculation can be found elsewhere [32]. The thickness of each sample was taken as the average value of five points measured before the experiment using a Mitutoyo 293–805 micrometer (Mitutoyo, Japan). FTIR spectra of the samples were measured using a Nicolet iS5 spectrometer (Thermo Fisher Scientific, USA) in attenuated total reflection mode using a Quest Specac accessory (400–4000 cm^−1^ spectral range, 32 scans, 2 cm^−1^ resolution).

### 4.5. Experimental Setup of Diffusion Dialysis Tests

Two series of tests were conducted in a continuous regime to study process parameter impacts on system performance in terms of free-acid yield, metal-ion rejection, and output stream composition. The first series consisted of tests performed at different ratios (*R*_V_) of acidic feed:demineralized water-inlet-volumetric flow in L.h^−1^ at laboratory temperature (20–25 °C). The second series consisted of tests performed at different feed temperatures (*T*_F_; from 20 °C to 40 °C) at fixed *R*_V_ (conditions summarized in Table 13).

Samples were collected from the outlet streams once the process had reached a steady-state. To check the state of the process, diffusate and the dialysate conductivity and flowrate were measured over time, with flowrate measured using the cylinder method (i.e., time needed to fill a cylinder with 100 mL of liquid).

**Figure 7:**
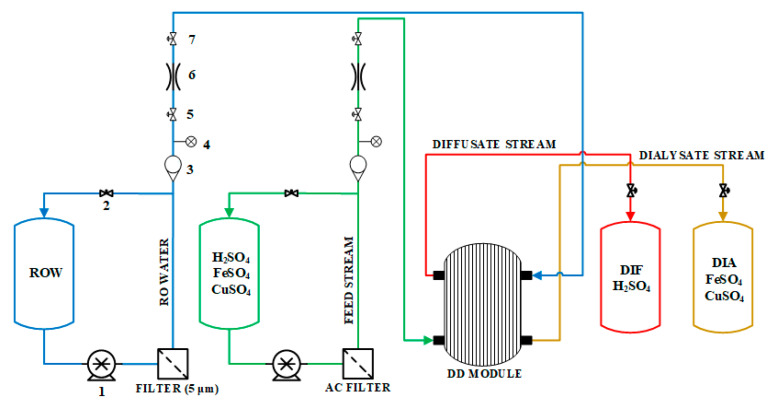
Simplified process flow diagram of the acid-recovery system. Instrumentation key: (1) pump; (2) needle valve; (3) rotameter; (4) pressure sensor; (5) manual valve; (6) flow control valve; (7) manual valve.

The experimental process consists of two parts, a hydraulic feed and diffusate and dialysate outlet streams (Figure 7). The hydraulic part consists of two Flojet pumps (Xylem Inc., USA), two filters (a 5 µm filter for the RO permeate and an activated carbon (AC) filter for the acidic feed stream) and a series of rotameters, pressure indicators and MARIC flow control valves (Maric Flow Control, Australia). Manual needle valves were located in the recirculation pipes to allow fine regulation of pressure and volumetric flow. High temperature levels (30 °C and 40 °C) were maintained in the feed solution by indirectly heating the salty acid barrel with a heating belt.

The diffusate and dialysate were collected in separate tanks. Samples from the outlet streams were collected directly from the sampling cock valves (i.e., not from the tanks) at pre-determined time intervals.

### 4.6. Calculations

Water uptake (*W*) was calculated by the following Equation (1):

(1) $$W=\frac{m_{wet}-m_{dry}}{m_{dry}}\times 100\%$$

where, *m*_wet_ and *m*_dry_ represent the membrane weight before and after dehydration.

The diffusion permeability coefficients (*P*_*s*_) was calculated using the following Equation (2):

(2) $$P_{s}=\frac{J_{s}d_{m}}{∆C_{s}}$$

where, $J_{s}$ is solute flow, $d_{m}$ is membrane thickness and $∆C_{s}$ is the solute concentration difference.

The area resistance (*R_area_*) and specific conductivity (σ) were calculated using the following Equations (3) and (4):

(3) $$R_{area}=\left(R_{cell+mem}-R_{cell}\right)\times S$$

(4) $$\sigma =\frac{d_{m}}{R_{area}}$$

where, $R_{cell+mem}$ and $R_{cell}$ represent cell resistance with and without the membrane and *S* is the membrane active area.

According to [34], Young’s modulus is calculated from the slope of the initial section of the stress-strain curve. The yield strength (transition from elasticity to plasticity) corresponded to the first local maximum of the 1-st order derivative of the stress-strain curve. In this case, tensile strength corresponds to maximum stress.

Volumetric ratio (*R_V_*) was defined according to the following Equation (5):

(5) $$R_{V}=\frac{{\dot{V}}_{FEED}\left(L.h^{-1}\right)}{{\dot{V}}_{ROW}\left(L.h^{-1}\right)}$$

where, ${\dot{V}}_{FEED}$ represents volumetric flow of the acid fed into the module and ${\dot{V}}_{ROW}$ represents volumetric flow of demineralized water.

Data evaluation was based on mass balance under steady-state conditions, as described in the following Equation (6):

(6) $$\sum^{\;}{\dot{m}}_{\mathrm{input}}-\sum^{\;}{\dot{m}}_{\mathrm{output}}=0$$

where, ${\dot{m}}_{input}\;\left({\dot{m}}_{output}\right)$ represents the mass flow of streams entering or leaving the system.

For the purpose of this study, Equation (6) has the form:

(7) $${\dot{m}}_{FEED}+{\dot{m}}_{ROW}={\dot{m}}_{DIA}+{\dot{m}}_{DIF}$$

Free-acid yield (*Y*) can then be calculated from either of the two following Equations (8) and (9):

(8) $$Y=\frac{{\dot{m}}_{H^{+},DIF}}{{\dot{m}}_{H^{+},FEED}}\times 100\%$$

(9) $$Y=\left(1-\frac{{\dot{m}}_{H^{+},DIA}}{{\dot{m}}_{H^{+},FEED}}\right)\times 100\%$$

In a steady-state, both Equations (8) and (9) are equivalent and can be derived from the mass balance of free acid in a steady state. The reported values represent average values of free-acid yield obtained from Equations (8) and (9).

Metal-ion rejection (*R*_i_) can also be calculated from the following two equivalent Equations (10) and (11):

(10) $$R_{i}=\frac{{\dot{m}}_{i,DIA}}{{\dot{m}}_{i,FEED}}\times 100\%$$

(11) $$R_{i}=\left(1-\frac{{\dot{m}}_{i,DIF}}{{\dot{m}}_{i,FEED}}\right)\times 100\%$$

where, *i* = Cu or Fe.

Reported values of metal-ion rejection for each test were calculated as average values from Equations (10) and (11).

Stream composition was used to evaluate stream ionic purity. The ionic purity (*P_j_*) of stream j was defined as follows (12):

(12) $$P_{j}=\frac{c_{H^{+},j}}{c_{H^{+},j}+\sum_{i}c_{i,j}}\times 100\%$$

where, *j* is FEED, DIF and DIA and *i* is Fe or Cu. *c_i,j_* is the concentration of the *i*-th compound in the *j*-th stream in ppm.

The total flux of acid $J_{H_{2}SO_{4}}$ was defined as the mass flow of acid ${\dot{m}}_{H_{2}SO_{4}}$ per effective membrane area (*S*):

(13) $$J_{H_{2}SO_{4}}=\frac{{\dot{m}}_{H_{2}SO_{4}}}{S}$$

Equations (8)–(13) are especially useful for evaluating the module’s separation performance, for comparing the degree of purification of recovered acid with inlet acidic feed or for scaling-up the industrial technology if a required flow of recoverable acid is provided.

### 4.7. Statistical Analysis

Measurement uncertainties for electrical conductivity and temperature were obtained from the manufacturer’s own documentation, while measurement uncertainties for heavy metal ion concentration and acidity were obtained by applying the Student’s t-distribution with a significance level of *p* < 0.05. Mass flow was characterized by a measurement uncertainty of 2%, obtained from the propagation-of-error formula.

## 5. Conclusions

A membrane-based separation process driven by a chemical-potential gradient was successfully used for the treatment of acidic wastewater and recovery of sulphuric acid, with different combinations of acidic feed and demineralized water volumetric-flow ratios influencing system performance in terms of sulphuric acid yield, metal ion rejection and component concentration in recovered-acid and reject streams. The temperature of the acidic feed stream increased mass transfer of both sulphuric acid and dissolved sulphate salts, with a significant effect observed at temperatures up to 30 °C.

A techno-economic study indicated that inclusion of DD into recovery of sulphuric acid from wastewater was viable and feasible, even for dilute solutions (5 wt.% sulphuric acid), with a simple payback period of ca 1 year. The payback period would be even shorter where more concentrated spent streams were generated.

A comparison of basic membrane (Fumasep^®^FAD-PET-75) physicochemical properties (i.e., ion exchange capacity, water uptake, thickness, permselectivity, acid and salt diffusion permeability, ionic conductivity, and stress-strain curves) before and after dialysis indicated only a slight drop in membrane selectivity and an increase in diffusion permeability. Furthermore, there were no significant changes in membrane IR spectra. Taken together, this indicates a relatively high chemical and mechanical membrane stability.

## Figures and Tables

**Table 1:** Summarized values for performance parameters used for Tests 1.1 and 2.1. Symbols: *Y*—yield of acid, *R*_Cu_^2+^—rejection of Cu^2+^, *R*_Fe_^2+^—rejection of Fe^2+^, $J_{H_{2}{\mathrm{SO}}_{4}}$—flux of acid, $w_{H_{2}{\mathrm{SO}}_{4},\;FEED\;\left(DIF/DIA\right)}$ —weight fraction of acid in feed (diffusate/dialysate) solution, *P*_FEED (DIF)_—ionic purity of feed (diffusate).

Parameter	Unit	Test 1.1	Test 2.1
*Y*	%	88 ± 5	93 ± 6
*R*_Cu_^2+^	%	95 ± 5	90 ± 10
*R*_Fe_^2+^	%	91 ± 9	86 ± 10
$J_{H_{2}{\mathrm{SO}}_{4}}$	g·m^−2^·h^−1^	81 ± 3	87 ± 3
$w_{H_{2}{\mathrm{SO}}_{4},\;FEED}$	%	5.18 ± 0.10	4.84 ± 0.10
$w_{H_{2}{\mathrm{SO}}_{4},\;DIF}$	%	4.39 ± 0.09	3.48 ± 0.07
$w_{H_{2}{\mathrm{SO}}_{4},\;DIA}$	%	0.66 ± 0.01	0.41 ± 0.01
*P*_FEED_	%	76.8 ± 1.3	76.0 ± 1.3
*P*_DIF_	%	97.5 ± 0.2	95.6 ± 0.3
$P_{\mathrm{DIF}}/P_{\mathrm{DIA}}$	-	3.2	4.4

**Table 2:** Flows of inlet and outlet streams (volumetric flows in L·h^−1^ and mass flows in kg·h^−1^). Symbols: ${\dot{V}}_{FEED\;\left(ROW/DIF/DIA\right)}$*—*volumetric flow rate of feed (RO permeate/diffusate/dialysate), ${\dot{m}}_{FEED\;\left(ROW/DIF/DIA\right)}$ —mass flow rate of feed (RO permeate/diffusate/dialysate).

Parameter	Test 1.1	Test 2.1
${\dot{V}}_{FEED}$	8.55 ± 0.05	9.42 ± 0.05
${\dot{V}}_{ROW}$	8.64 ± 0.05	11.45 ± 0.05
${\dot{V}}_{DIF}$	8.98 ± 0.09	12.28 ± 0.12
${\dot{V}}_{DIA}$	8.21 ± 0.08	8.58 ± 0.09
${\dot{m}}_{FEED}$	8.83 ± 0.05	9.70 ± 0.05
${\dot{m}}_{ROW}$	8.63 ± 0.05	11.42 ± 0.05
${\dot{m}}_{DIF}$	9.22 ± 0.18	12.53 ± 0.25
${\dot{m}}_{DIA}$	8.24 ± 0.16	8.59 ± 0.17

**Table 3:** Analytical measurements for Tests 1.1 and 2.1.

Composition	Unit	Feed	Diffusate	Dialysate	Feed	Diffusate	Dialysate
		Test 1.1			Test 2.1		
H_2_SO_4_	g·L^−1^	53.5 ± 1.1	45.0 ± 0.9	6.6 ± 0.1	50.0 ± 1.0	35.5 ± 0.7	4.1 ± 0.1
Fe^2+^	ppm	284 ± 14	21 ± 1	263 ± 13	282 ± 14	30 ± 2	263 ± 13
Cu^2+^	ppm	50 ± 3	3.2 ± 0.2	50 ± 3	43 ± 2	3.4 ± 0.2	42 ± 2
Density	kg·L^−1^	1.033± 0.001	1.026 ± 0.001	1.004 ± 0.001	1.030 ± 0.001	1.020 ± 0.001	1.001 ± 0.001

**Table 4:** Summarized values of performance parameters used for Tests 1.2, 2.2, and 3.2. Symbols: *T*_FEED_—temperature of feed, *Y*—yield of acid, *R*_Cu_^2+^—rejection of Cu^2+^, *R*_Fe_^2+^—rejection of Fe^2+^, $J_{H_{2}{\mathrm{SO}}_{4}}$—flux of acid, $w_{H_{2}{\mathrm{SO}}_{4},\;FEED\;\left(DIF/DIA\right)}$ —weight fraction of acid in feed (diffusate/dialysate) solution, *P*_FEED (DIF)_—ionic purity of feed (diffusate).

Parameter	Unit	Test 1.2	Test 2.2	Test 3.2
*T*_FEED_	°C	20–25	30	40
*Y*	%	75 ± 4	76 ± 4	76 ± 4
*R*_Cu_^2+^	%	96 ± 4	95 ± 5	94 ± 6
*R*_Fe_^2+^	%	96 ± 4	95 ± 5	93 ± 7
$J_{H_{2}{\mathrm{SO}}_{4}}$	g·m^−2^·h^−1^	66 ± 3	71 ± 3	72 ± 3
$w_{H_{2}{\mathrm{SO}}_{4},\;FEED}$	%	4.8 ± 0.1	4.9 ± 0.1	4.9 ± 0.1
$w_{H_{2}{\mathrm{SO}}_{4},\;DIF}$	%	4.4 ± 0.1	4.4 ± 0.1	4.6 ± 0.1
$w_{H_{2}{\mathrm{SO}}_{4},\;DIA}$	%	1.30 ± 0.02	1.19 ± 0.02	1.25 ± 0.03
*P*_FEED_	%	75.7 ± 1.6	76.0 ± 1.3	75.8 ± 1.3
*P*_DIFF_	%	98.2 ± 0.1	97.6 ± 0.2	97.2 ± 0.2
$P_{\mathrm{DIF}}/P_{\mathrm{DIA}}$	-	2.2	2.3	2.2

**Table 5:** Inlet and outlet stream flows (volumetric flows in L·h^−1^ and mass flows in kg·h^−1^) for Tests 1.2, 2.2 and 3.2. Symbols: ${\dot{V}}_{FEED\;\left(ROW/DIF/DIA\right)}$*—*volumetric flow rate of feed (RO permeate/diffusate/dialysate), ${\dot{m}}_{FEED\;\left(ROW/DIF/DIA\right)}$ —mass flow rate of feed (RO permeate/diffusate/dialysate).

Parameter	Test 1.2	Test 2.2	Test 3.2
${\dot{V}}_{FEED}$	9.04 ± 0.05	9.18 ± 0.05	9.36 ± 0.05
${\dot{V}}_{ROW}$	6.93 ± 0.05	7.23 ± 0.05	6.95 ± 0.05
${\dot{V}}_{DIF}$	7.36 ± 0.07	7.59 ± 0.08	7.51 ± 0.08
${\dot{V}}_{DIA}$	8.59 ± 0.09	8.82 ± 0.09	8.81 ± 0.09
${\dot{m}}_{FEED}$	9.32 ± 0.05	9.46 ± 0.05	9.65 ± 0.05
${\dot{m}}_{ROW}$	6.91 ± 0.05	7.22 ± 0.05	6.94 ± 0.05
${\dot{m}}_{DIF}$	7.57 ± 0.15	7.80 ± 0.16	7.72 ± 0.15
${\dot{m}}_{DIA}$	8.66 ± 0.17	8.88 ± 0.18	8.87 ± 0.18

**Table 6:** Analytical measurements for Test 3.2.

Composition	Unit	Feed	Diffusate	Dialysate
H_2_SO_4_	g·L^−1^	50.4 ± 1.0	47.5 ± 0.9	12.6 ± 0.3
Fe^2+^	ppm	286 ± 14	25 ± 1	282 ± 14
Cu^2+^	ppm	45 ± 2	3.7 ± 0.2	45 ± 2
Density	kg·L^−1^	1.031 ± 0.001	1.028 ± 0.001	1.008 ± 0.001

**Table 7:** Physical and chemical parameters of Fumasep^®^ FAD-75.

Parameter	Unit	Value (Initial)	Value (after Use)
Thickness (dry)	μ	76 ± 1	78 ± 1
Ion exchange capacity (Cl^−^ form)	meq·g^−1^	1.51 ± 0.03	1.53 ± 0.03
Specific conductivity in SO_4_^−^ form	mS·cm^−1^	22 ± 2	15 ± 1
Permselectivity (at 0.1/0.5 mol·kg^−1^ NaCl)	%	92.2 ± 0.4	90.8 ± 0.4
Water uptake	wt.%	28 ± 1	26 ± 1
Young’s modulus	MPa	750 ± 30	740 ± 30
Yield strength	MPa	8.5 ± 0.5	9.4 ± 0.5
Tensile strength	MPa	42 ± 1	55 ± 1
Elongation at break	%	19 ± 2	22 ± 2
CuSO_4_ diffusion permeability coefficient at 0.1M CuSO_4_	cm^2^·s^−1^	(3.0 ± 0.2)·10^−7^	(3.7 ± 0.2)·10^−7^
H_2_SO_4_ diffusion permeability coefficient at 0.1M H_2_SO_4_	cm^2^·s^−1^	(3.8 ± 0.2)·10^−6^	(4.6 ± 0.2)·10^−6^

**Table 8:** Unit costs of the considered sources. The price of electricity reflects the situation in the Czech Republic.

Item	Unit	Unit Cost
Electricity	USD/kWh	0.1
Demineralized water	USD/m^3^	10
96% H_2_SO_4_	USD/t	400
90% Ca(OH)_2_	USD/t	120
Solid waste disposal	USD/t	300

**Table 9:** Annual expenses for each case of the economic study. (A1) processing 5% H_2_SO_4_ wastewater without DD; (B1) processing 5% H_2_SO_4_ wastewater with DD; (A2) processing 10% H_2_SO_4_ wastewater without DD; (B2) processing 10% H_2_SO_4_ wastewater with DD.

Case	Unit	A1	B1	A2	B2
Slaked lime 90%	USD	4230	1083	8444	2151
Sludge disposal	USD	50,153	12,846	100,121	25,508
Electricity	USD	n/a	792	n/a	792
Sulphuric acid 96%	USD	52,703	39,635	52,606	26,470
Water	USD	8530	7490	9927	7848
Total	USD	115,616	61,846	171,098	62,769

**Table 10:** Annual cash flow for each case and expected payback period for DD investment.

Item	Unit	A1	B1	A2	B2
Expenses	USD	115,616	61,846	171,098	62,769
Savings	USD	0	53,770	0	108,329
Payback period	year	-	0.97	-	0.48

**Table 11:** Average composition of feed solution.

Composition	Unit	Feed Stream
H_2_SO_4_	g·L^−1^	51 ± 1
Fe^2+^	ppm	283 ± 14
Cu^2+^	ppm	45 ± 2
Electrical conductivity	mS·cm^−1^	221.5 ± 1.1
Density	kg·L^−1^	1.031 ± 0.001

**Table 12:** Spiral-wound module characteristics. Parameters were downloaded from the online module datasheet [30].

Parameter	Value
Flow	5–15 L·h^−1^ each channel
Pressure loss	80 mbar (at 5 L·h^−1^)–400 mbar (at 15 L·h^−1^)
Operating pressure	0.1–1.5 bar (g)
Differential pressure	<200 mbar (between the channels)
Operating temperature	5 °C–40 °C
Empty weight	ca. 8 kg
Fill volumes	ca. 4.5 L each channel

**Table 13:** Conditions for Series 1 and Series 2 tests. Symbols: *R*_V_—volumetric flow rate ratio (feed/RO permeate), *T*_F_—temperature of the inlet feed.

Parameter	Unit	Test 1.1	Test 2.1	Test 1.2	Test 2.2	Test 3.2
*R*_V_	-	9/9	9/11	9/7	9/7	9/7
*T*_F_	°C	20–25	20–25	20–25	30	40

## Data Availability

The data presented in this study are available on request from the corresponding author.

## Abbreviations

AC	Activated carbon
AEMs	Anion-exchange membranes
BPPO	Bromomethylated poly (phenylene oxide)
DD	Diffusion dialysis
DIA	Dialysate
DIF	Diffusate
FEED	Salty acid fed to a module
PET	Polyethylene terephthalate
R.H.	Relative humidity
RO	Reverse osmosis
ROW	Permeate of reverse osmosis
SEM	Scanning electron microscope
USD	United States dollar
